# Supplementary material for: The role of antifreeze glycoprotein (AFGP) and polyvinyl alcohol/polyglycerol (X/Z-1000) as ice modulators during partial freezing of rat livers
Source: Front Phys. Author manuscript; Available in PMC 2023 May 5. (PMC10161798; doi:10.3389/fphy.2022.1033613)
Supplement: Supplementary Materials [file NIHMS1869068-supplement-Supplementary_Materials.docx]

**SUPPLEMENTAL FIGURES AND TABLES**

**Supplemental Figure S1:** Potassium levels in partially frozen livers. (**A**) Comparing perfusate potassium levels in partially frozen rat livers stored at -10°C (n = 4) vs –15°C (n = 9) during SNMP revealed no differences between the groups. (**B**) Comparing perfusate potassium in partially frozen rat livers with AFGP (n=4) or with X/Z-1000 ice modulators (n=4) versus 12% glycerol control (n=13) also showed no differences.


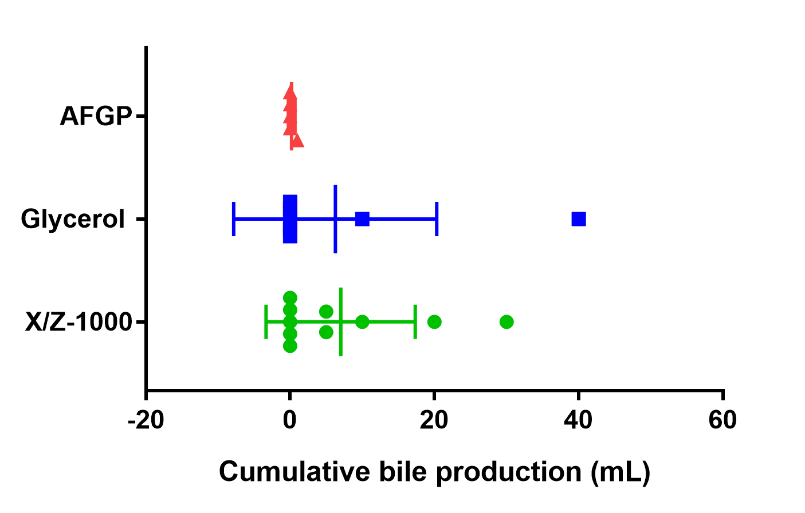


**Supplemental Figure S2.** Cumulative bile production of rat livers partially frozen with AFGP (n=5), X/Z-1000 (n=10), and 12% glycerol controls (n=8) after 3 hours of recovery with SNMP. Mean with standard deviation and individual data points are shown.

**Supplemental Table S1:** Composition of all solutions used in each phase of the partial freezing protocol. WE = Williams E, UW = University of Wisconsin, PEG = polyethylene glycol, BSA = bovine serum albumin, HES = hydroxyethyl starch, 3-OMG = 3-O-methyl-D-glucose, CPA = cryoprotective agent, INA = ice nucleating agents, U/l = Units per liter, *present in UW solution.

|  | **Subnormothermic  Preconditioning solution** | **Hypothermic  Preloading solution** | **Storage  Solution** | **Thawing Solution** | **Subnormothermic Recovery  Solution** |
| --- | --- | --- | --- | --- | --- |
| ***Base solution*** | **WE** | **WE** | **UW** | **WE** | **WE** |
| ***Total volume*** | **250 ml** | **250 ml** | **100 ml** | **250 ml** | **500 ml** |
|  |  |  |  |  |  |
| ***Additives*** |  |  |  |  |  |
| Insulin | 200 U/l | 200 U/l | 10 U/l | 10 U/l | 20 U/l |
| Heparin | 10,000 U/l | 4,000 U/l | --- | 1,000 U/l | 1,000 U/l |
| Dexamethasone | 24 mg/l | 24 mg/l | 24 mg/l | 24 mg/l | 24 mg/l |
| Hydrocortisone | 25 mg/ml | 25 mg/ml | --- | 25 mg/ml | 25 mg/ml |
| Penicillin | 40,000 ug/l | 40,000 ug/l | --- | 40,000 ug/l | 40,000 ug/l |
| Streptomycin | 40,000 U/l | 40,000 U/l | --- | 40,000 U/l | 40,000 U/l |
| Glutathione | --- | --- | 0.922 g/l* | 1.536 g/l | 1.536 g/l |
|  |  |  |  |  |  |
| ***Macromolecules*** |  |  |  |  |  |
| 35 kDA PEG | 20 g/l | 20 g/l | 50 g/l | 20 g/l | 20 g/l |
| BSA | 50 g/l | 50 g/l | --- | 50 g/l | 50 g/l |
| HES | --- | 30 g/l | 50 g/l* | 30 g/l | --- |
|  |  |  |  |  |  |
| ***Saccharides*** |  |  |  |  |  |
| 3-OMG | 19.42 g/l | 19.42 g/l | --- | 19.42 g/l | --- |
| Raffinose | --- | 15.12 g/l | 17.83 g/l* | 15.12 g/l | --- |
| Trehalose | --- | --- | 18.92 g/l | 18.92 g/l | --- |
|  |  |  |  |  |  |
| ***CPA / INA*** |  |  |  |  |  |
| Glycerol | --- | 60 ml/l | 120 ml/l | --- | --- |
| Snomax | --- | --- | 1 g/l | --- | --- |

**Supplemental Table S2: List of suppliers for each reagent used in partial freezing solutions.**

| ***Reagent*** |  | ***Supplier*** |
| --- | --- | --- |
| University of Wisconsin (UW) solution |  | Bridge to Life |
| Williams' Medium E |  | Sigma-Aldrich |
| Insulin (Humulin R) |  | MGH pharmacy |
| Sodium heparin |  | MGH pharmacy |
| Dexamethasone |  | Sigma-Aldrich |
| Hydrocortisone |  | MGH pharmacy |
| Penicillin-Streptomycin |  | Invitrogen |
| L-Glutathione |  | Sigma-Aldrich |
| Bovine Serum Albumin (BSA) |  | Sigma-Aldrich |
| 35kDa Polyethylene glycol (PEG) |  | Sigma-Aldrich |
| 3–O–methyl glucose |  | Chem-Impex |
| D-(+)-Trehalose dihydrate |  | Sigma-Aldrich |
| Glycerol |  | Fisher Scientific |
| Antifreeze glycopeptides (AFGP) |  | A/F Protein Inc. |
| X/Z-1000 |  | 21st Century Medicine |
| Snomax |  | Telemet |
